# Metagenomes from Thawing Low-Soil-Organic-Carbon Mineral Cryosols and Permafrost of the Canadian High Arctic

**DOI:** 10.1128/genomeA.01217-14

**Published:** 2014-11-20

**Authors:** Archana Chauhan, Alice C. Layton, Tatiana A. Vishnivetskaya, Daniel Williams, Susan M. Pfiffner, Bhanu Rekepalli, Brandon Stackhouse, Maggie C. Y. Lau, Tommy J. Phelps, Nadia Mykytczuk, Jennifer Ronholm, Lyle Whyte, Tullis C. Onstott, Gary S. Sayler

**Affiliations:** a Center for Environmental Biotechnology, University of Tennessee, Knoxville, Tennessee, USA; b Joint Institute for Biological Sciences, University of Tennessee, Knoxville, and Oak Ridge National Laboratory, Knoxville, Tennessee, USA; c Department of Microbiology, University of Tennessee, Knoxville, Tennessee, USA; d Department of Earth and Planetary Sciences, University of Tennessee, Knoxville, Tennessee, USA; e National Institute for Computational Sciences, University of Tennessee, Knoxville, and Oak Ridge National Laboratory, Oak Ridge, Tennessee, USA; f Department of Geosciences, Princeton University, Princeton, New Jersey, USA; g Biosciences Division, Oak Ridge National Laboratory, Oak Ridge, Tennessee, USA; h Vale Living with Lakes Centre, Laurentian University, Sudbury, Ontario, Canada; i Department of Natural Resource Sciences, Macdonald Campus of McGill University, Ste. Anne de Bellevue, Quebec, Canada

## Abstract

Microbial release of greenhouse gases from thawing permafrost is a global concern. Seventy-six metagenomes were generated from low-soil-organic-carbon mineral cryosols from Axel Heiberg Island, Nunavut, Canada, during a controlled thawing experiment. Permafrost thawing resulted in an increase in anaerobic fermenters and sulfate-reducing bacteria but not methanogens.

## GENOME ANNOUNCEMENT

Terrestrial ecosystems are major components of both the carbon and nitrogen cycles contributing to atmospheric gas concentrations (1). Permafrost thawing followed by microbial decomposition of previously frozen organic C is of concern because it may lead to a positive feedback loop of emitted CO_2_, CH_4_, and N_2_O from high-latitude terrestrial ecosystems to the atmosphere. To date, most studies have focused on this effect in organic carbon-rich cryosols. Less information is available regarding microbial activity and the potential for CH_4_ generation in soil-organic-carbon (SOC)/nitrogen-poor mineral cryosols, such as those found on Axel Heiberg Island, Nunavut, Canada, at the McGill Arctic Research Station. The goal of this project was to monitor microbial community changes during 18 months of controlled thawing under varying light exposure and water saturation conditions of 17 one-meter-long intact cores consisting of active layer and permafrost soils and correlate these changes with temporal and vertical changes in the aqueous geochemical, isotopic compositions, and CO_2_ and CH_4_ gas flux (M. C. Y. Lau, B. T. Stackhouse, A. C. Layton, A. Chauhan, T. A. Vishnivetskaya, K. Chourey, N. C. S. Mykytczuk, P. C. Bennett, G. Lamarche-Gagon, N. Burton, J. Ronholm, W. H. Pollard, C. R. Omelon, D. M. Medvigy, R. L. Hettich, S. M. Pfiffner, L. G. Whyte, T. C. Onstott, submitted for publication; B. T. Stackhouse, T. A. Vishnivetskaya, A. C. Layton, A. Chauhan, S. M. Pfiffner, N. C. S. Mykytczuk, L. G. Whyte, L. Hedin, N. Saad, T. C. Onstott, submitted for publication).

As part of the thawing experiment, cores were subsampled for metagenomic analyses. Total community genomic DNA was extracted using Fast DNA SPIN Kit (MP Biomedicals, Irvine, CA) (2). Metagenomic libraries were prepared using the Illumina Nextera DNA library preparation kit (Illumina, Inc., San Diego, CA) followed by 2 × 100 bp sequencing on Illumina HiSeq 2000 platform generating 647 Gb of metagenomic data. Seventy-six metagenomes spanning 5 treatment conditions, 4 depths (5 cm, root zone; 35 cm, active layer above water table; 65 cm, active layer below water table; and 80 cm, permafrost), and 5 time points (0 to 18 months) were annotated in MG-RAST version 3.3.7 (3).

At time 0 across all depths, bacteria were the dominant domain (87.7% ± 6.2%) followed by eukaryota (11.2% ± 6.2%). Archaea and viruses comprised only 0.9% ± 0.6% and 0.1% ± 0.1% of the sequences, respectively. The largest changes in the microbial community structure were related to depth with *Proteobacteria* and *Actinobacteria* being the most abundant phyla. *Proteobacteria* decreased in abundance between the upper 5 cm (22.1% ± 0.9%) and permafrost 80 cm (13.6% ± 4.6%), while *Actinobacteria* increased in abundance between the upper 5 cm (33.1% ± 4.0%) and permafrost 80 cm (54.0% ± 3.1%). Over the course of 18 months, the microbial community structure remained remarkably constant in the upper 5-cm soil samples, with changes observed at increasing depths. Genera involved in anaerobic fermentation (*Clostridium*) and sulfite/sulfate reduction (family *Peptococcaceae*: *Desulfosporosinus, Desulfitobacterium*, and *Desulfotomaculum*) increased at 65 cm and 80 cm with the thawing period. In contrast to metagenome data generated from thawed/unthawed permafrost samples from other locations (4, 5), methanogens were not abundant in either the initial core samples or after prolonged thawing. This lesser abundance may be related to the lesser SOC and greater sulfate concentrations and sulfate-reducing activity.

These metagenomic data sets will augment the detailed chemical measurements collected during the core thawing experiment and provide insight into metabolic changes that may occur during the warming of low-SOC mineral cryosols in this Arctic region.

### Nucleotide sequence accession number.

Nucleotide sequences obtained were deposited at the NCBI Sequence Read Archive under the accession number SRP047512.
